# Transcriptomic Analysis of Short-Term Salt Stress Response in Watermelon Seedlings

**DOI:** 10.3390/ijms21176036

**Published:** 2020-08-21

**Authors:** Qiushuo Song, Madhumita Joshi, Vijay Joshi

**Affiliations:** 1 Department of Horticultural Sciences, Texas A&M University, College Station, TX 77843, USA; qiushuo1995@gmail.com; 2 Texas A&M AgriLife Research and Extension Center, Uvalde, TX 78801, USA; Madhumita.Joshi@ag.tamu.edu

**Keywords:** watermelon, salt stress, RNA-seq, amino acids, endocytosis

## Abstract

Watermelon (*Citrullus lanatus* L.) is a widely popular vegetable fruit crop for human consumption. Soil salinity is among the most critical problems for agricultural production, food security, and sustainability. The transcriptomic and the primary molecular mechanisms that underlie the salt-induced responses in watermelon plants remain uncertain. In this study, the photosynthetic efficiency of photosystem II, free amino acids, and transcriptome profiles of watermelon seedlings exposed to short-term salt stress (300 mM NaCl) were analyzed to identify the genes and pathways associated with response to salt stress. We observed that the maximal photochemical efficiency of photosystem II decreased in salt-stressed plants. Most free amino acids in the leaves of salt-stressed plants increased many folds, while the percent distribution of glutamate and glutamine relative to the amino acid pool decreased. Transcriptome analysis revealed 7622 differentially expressed genes (DEGs) under salt stress, of which 4055 were up-regulated. The GO analysis showed that the molecular function term “transcription factor (TF) activity” was enriched. The assembled transcriptome demonstrated up-regulation of 240 and down-regulation of 194 differentially expressed TFs, of which the members of ERF, WRKY, NAC bHLH, and MYB-related families were over-represented. The functional significance of DEGs associated with endocytosis, amino acid metabolism, nitrogen metabolism, photosynthesis, and hormonal pathways in response to salt stress are discussed. The findings from this study provide novel insights into the salt tolerance mechanism in watermelon.

## 1. Introduction

Soil salinization is recognized as a major problem for agricultural production and sustainability at a global level. The area of degraded saline soils has rapidly increased due to climate change and limited rainfall, posing a great challenge to global food security [1]. It is estimated that around 20% to 50% of irrigated land is salt-affected in arid and semi-arid regions [2,3,4]. Salt adversely impacts plant growth and development as it holds water and nutrients in the soil at high tension making these components unavailable for plants at the root zone. 

Watermelon (*Citrullus lanatus* L.) is a widely popular vegetable fruit crop for human consumption worldwide. Although China tops the watermelon production, it is grown widely across arid and semi-arid environments in the world. Watermelons can tolerate some degree of soil acidity [5] but grow best in non-saline sandy loam or silt loam soils. The research efforts to improve salt tolerance using conventional or transgenic breeding have had limited success due to the genetically and physiologically complex nature of salt-induced responses [3,6]. In watermelon, approaches such as salt-tolerant rootstocks [5,7,8] and agronomical practices [9,10] have been explored. Understanding the molecular mechanisms underscoring the salt stress response in model xerophyte plant species would contribute to finding conserved response cues in plants and identifying salt-tolerant traits [11,12,13]. Watermelon, being relatively tolerant of drought and salt stress, makes an excellent model crop to study salt stress-induced responses. There has been little research on transcriptome analysis to understand molecular regulation of salt stress-induced responses in watermelon. In the present study, we examined gene expression changes in watermelon seedlings due to short-term salt stress using RNA sequencing (RNA-seq) and discuss putative candidate genes and pathways associated with salt stress induced responses. The results from this study will provide a foundation to understand salt tolerance mechanisms and its exploitation to allow development of salt-tolerant watermelon cultivars.

## 2. Results

### 2.1. Validation of Salt Stress Treatment 

**Figure 1:**
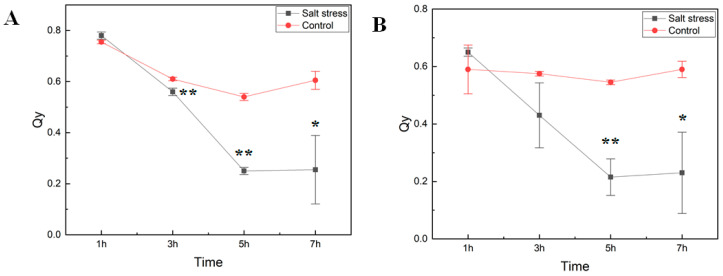
Photosynthetic efficiency of photosystem II. The maximal quantum yield of photosystem II (PSII) photochemistry (Fv/Fm) (**A**) and the efficiency of excitation capture of the open PSII center (Fv’/Fm’) (**B**) in *cv*. Crimson Sweet leaves under salt stress were measured using FluorPen (PAR-FluorPen FP 110/D). Asterisks ** and * represent significant difference at *p* < 0.05 and *p* < 0.1, respectively. Qy, equal to Fv/Fm in the dark (**A**) or light-adapted (**B**) samples, photosystem II efficiency. The error bars represent the standard deviation.

We studied how short-term exposure of watermelon seedlings to salt stress changed chlorophyll fluorescence parameters (as determined on dark-adapted and illuminated leaves) and amino acid metabolism. Six-week-old seedlings of the cultivar Crimson Sweet subjected to salt stress treatment were monitored for photosystem II (PSII) performance to measure the maximal quantum yield of PSII photochemistry (Fv/Fm) and the efficiency of excitation capture of the open PSII center (Fv’/Fm’) (Figure 1). Measurements of photosystem II efficiency showed a consistent reduction in the PSII efficiency (Qy) in both dark-adapted and illuminated leaf samples. The decrease in Qy in treated plants was much more rapid than the control ones over time. Early decrease in Qy in our experiment is consistent with reports showing inhibition of PSII activity due to salt stress [8,14,15]. In the light-adapted leaves, Qy of salt-stressed tissues was dramatically lower than that of control plants, confirming a negative effect on the quantum yield of PSII electron transport by salinity stress after seven hours of exposure.

**Figure 2:**
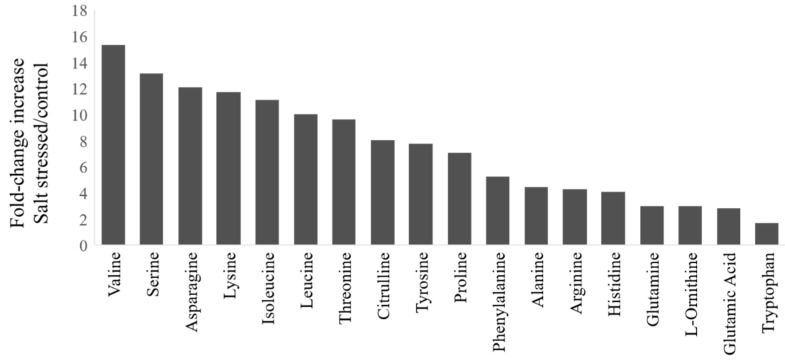
Fold-change increases in amino acids in watermelon leaves due to salt stress: The fold-change in plants treated with NaCl relative to the control group for amino acids showing significant changes (*t*-test, *p* < 0.05). The absolute amino acid quantities were normalized using internal standards and expressed as fold-change relative to control.

During salt stress, plants accumulate high concentrations of compatible osmolytes, such as nitrogen-containing compounds, mainly amino acids. In this study, most free amino acids showed several-fold increases in response to salt stress implying that the accumulation of free amino acids is crucial to salt stress in watermelon (Figure 2). The changes in the percent of amino acids relative to the pool size are shown in Figure S1 (Supplementary Materials) and the absolute amounts of each amino acid in Table S1 (Supplementary Materials). The branched-chain amino acids (valine, Val; isoleucine, Ile; leucine, Leu), serine (Ser), and Asparagine (Asn) showed much higher fold change increases than glutamate (Glu) and glutamine (Gln). The relative proportion of most amino acids also increased in the leaves after salt stress, except Glu, Gln, tryptophan (Trp), and glycine (Gly). No significant changes were observed in the content of most amino acids in roots due to short-term salt stress (Table S1), implying their limited localized synthesis or transport. It has been suggested that high abundant amino acids (proline, Pro; Arginine, Arg; asparagine, Asn, Glu) are synthesized during abiotic stress, while the low abundant amino acids (BCAAs) accumulate due to increased protein turnover or degradation under conditions such as salt stress [16,17]. However, such an increase in amino acid accumulation through proteolysis happens to a lesser extent in salt stress than drought stress [18]. Consistent with the meta-analysis [1], we also observed a significant decrease in the proportion of Glu and Gln, which serve as precursors for the synthesis of Pro, citrulline (Cit), and Arg, as well as polyamines. There was a limited increase in terms of fold change or change in percent accumulation in Pro or Cit, which are a major drought stress-induced amino acids in watermelon leaves [19]. Our results are consistent with studies that showed non-overlapping patterns of amino acid accumulation in salt stress and drought stress as well as a smaller increase in Cit due to salt stress [20] than drought stress. 

### 2.2. Transcriptome Profiling of Salt-Stressed Seedlings 

To understand the transcriptomic changes due to salt stress, we performed RNA-seq analysis of seedlings exposed to salt stress. A total of six libraries were sequenced using the Illumina HiSeq platform comprising three replicates each of the control and salt-treated plants. On average, 42.20 to 47.67 million paired-end raw reads were generated from leaf tissues in both treatments, of which more than 96% mapped to the reference watermelon genome (Supplementary Materials, Table S2). The RNA-seq dataset is accessible through GEO Series accession number GSE146087 (https://www.ncbi.nlm.nih.gov/geo/).

### 2.3. Identification of Differentially Expressed Genes (DEGs)

**Figure 3:**
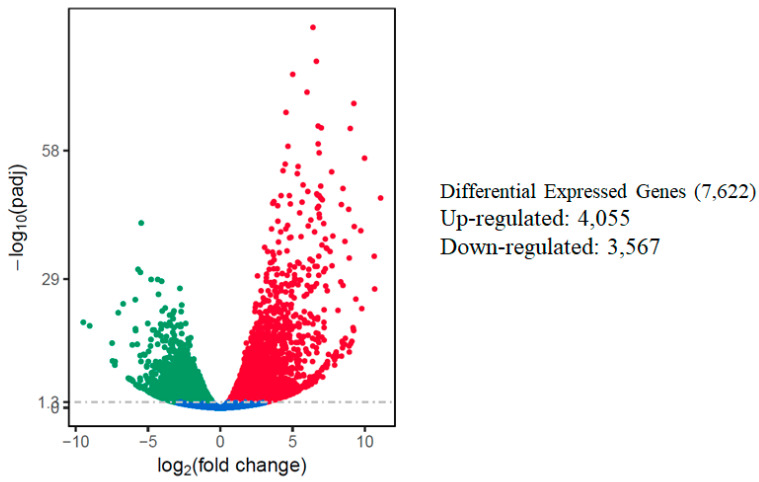
Summary of differentially expressed genes (DEGs) in the watermelon leaves during salt stress. Each point represents a gene; blue dots indicate no significant difference; red dots indicate up-regulated DEGs; green dots indicate down-regulated DEGs. The horizontal axis shows the fold change of genes between different samples (padj < 0.05), and the vertical coordinate indicates the statistically significant degree of changes in gene expression levels at −log10 (padj *p*-value).

The relative expression levels of genes were evaluated as the fragment per kilobase of transcript sequence per millions base pairs sequenced (FPKM) values, calculated based on the uniquely mapped reads for under control or salt stress condition. The RNA-seq data identified a total of 7622 differentially expressed genes in response to salt stress using comparative analysis when a cutoff of adjusted *p*-value (padj) < 0.05 and |log2fold change [L2fc]| > 1 were used. Out of the total DEGs, 4055 (53.2%) of those DEGs were up-regulated, while 3,567 (46.8%) of them were down-regulated. A volcano scatter plot showing the number of DEGs (Figure 3) and a list of DEGs is presented in the Supplementary Materials (Table S3). A wider dispersion indicates the presence of a higher level of difference regarding gene expression in response to salt stress. A higher number of up-regulated than down-regulated DEGs is consistent with a meta-analysis that included 25 independent salt stress transcriptomic studies [12], suggesting activation of a set of conserved genes regulating intrinsic salt-stress induced responses. 

### 2.4. GO, and KEGG Enrichment Results of DEGs

**Figure 4:**
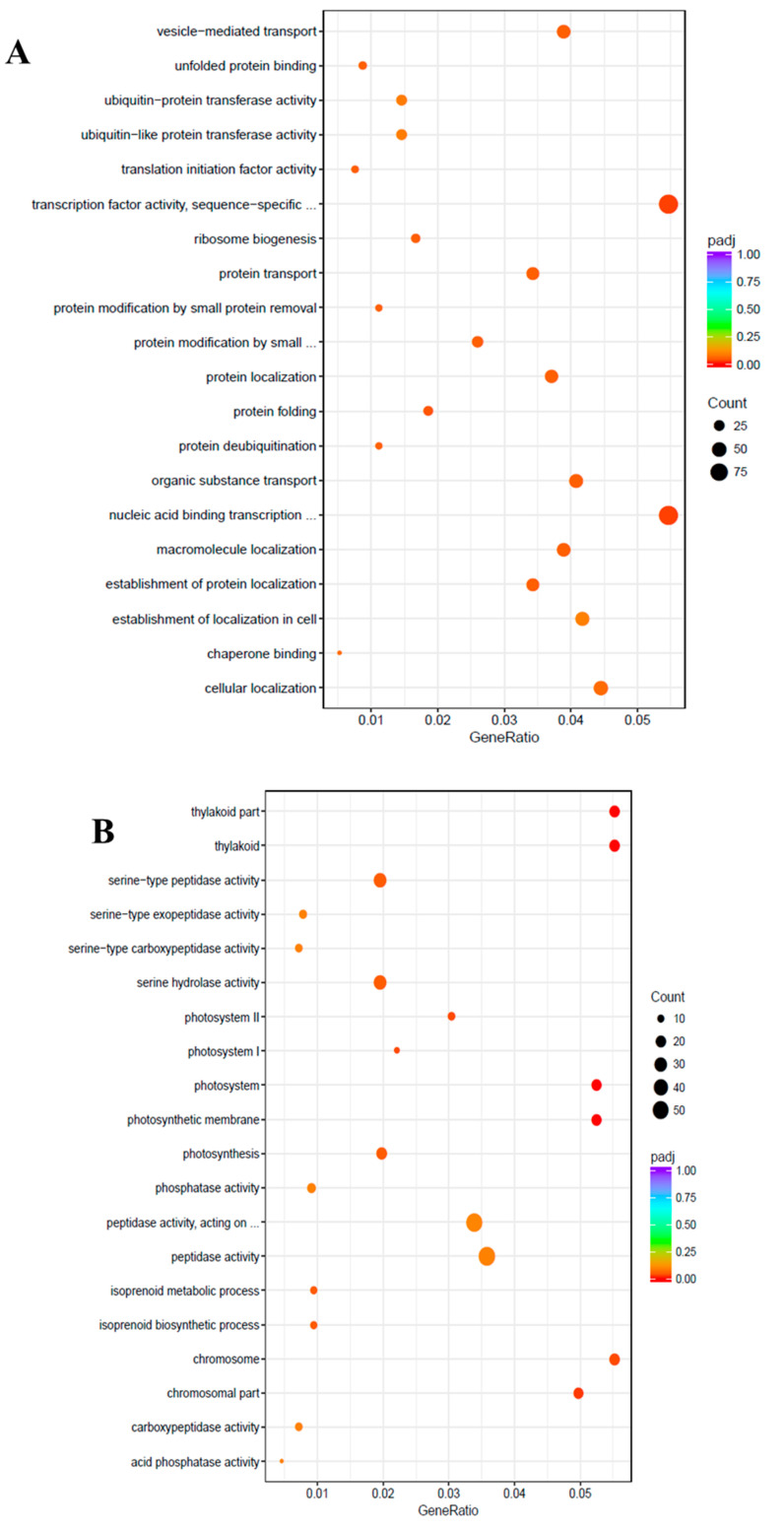
Gene Ontology (GO) enrichment scatter plot. The GO enrichment analysis showing the top 20 enriched functions for up-regulated (**A**) and down-regulated (**B**) DEGs. The horizontal axis is GeneRatio (the ratio between the number of differentially expressed genes in each GO term, and all differentially expressed genes that can be found in the GO database). The vertical axis is the description of GO terms. The significance showing padj *q*-values are shown as a color scale, where the color and size of the dots represent the range of *q*-value, and the number of DEGs mapped to the indicated functions, respectively.

To uncover the molecular mechanisms underlying the salt tolerance in watermelon leaves, the DEGs were characterized using the Gene Ontology (GO) knowledgebase (http://geneontology.org/). GO enrichment scatterplots show the top 20 enriched functions for up- or down-regulated DEGs due to salt stress (Figure 4). Among the biological process (BP) terms the function “protein folding” (GO:0006457) and among the molecular function (MF) the function “transcription factor activity”, sequence-specific DNA binding (GO:0003700) were enriched in the up-regulated unigenes with the cutoff of adjusted *q*-value < 0.05. On the contrary, in the cellular component (CC) category, “thylakoid” (GO:0009579), “photosystem” (GO:0009521), “photosynthetic membrane” (GO:0034357), “chromosomal part” (GO:0044427), “photosystem I” (GO:0009522), “chromosome” (GO:0005694), and “photosystem II” (GO:0009523) were the most enriched GO terms among the down-regulated DEGs.

**Figure 5:**
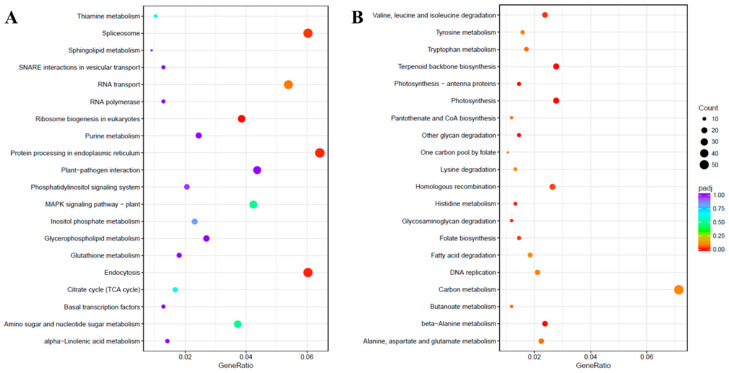
Kyoto Encyclopedia of Genes and Genomes (KEGG) enrichment scatter plot. The KEGG enrichment analysis showing the top 20 enriched pathways for up-regulated (**A**) and down-regulated (**B**) DEGs. The horizontal axis is GeneRatio (the ratio between the number of differentially expressed genes in each pathway, and all differentially expressed genes that can be found in the KEGG database). The vertical axis is the description of the KEGG term. The significance showing padj *q*-values are shown as a color scale, where the color and size of the dots represent the range of *q*-value and the number of DEGs mapped to the individual pathways, respectively.

Pathway analysis of DEGs was performed using the Kyoto Encyclopedia of Genes and Genomes (KEGG) pathway database with KOBAS [21]. The up-regulated and down-regulated DEGs were assigned to 106 and 114 pathways, respectively. The KEGG enrichment analysis showing the top 20 enriched functions is shown in Figure 5. The KEGG pathway annotations like “Ribosome biogenesis in eukaryotes”, “Endocytosis”, “Protein processing in endoplasmic reticulum” and “Spliceosome” were enriched in the up-regulated unigenes due to salinity stress. However, among the down-regulated unigenes, several KEGG pathways were significantly enriched such as “glycan degradation”, “beta-Alanine metabolism”, “Terpenoid backbone biosynthesis”, “Photosynthesis”, “Photosynthesis-antenna proteins”, “Histidine metabolism”, “Glycosaminoglycan degradation”, “Valine, leucine, and isoleucine degradation”, “Folate biosynthesis” and “Homologous recombination”. 

### 2.5. Differentially Expressed Transcription Factors (TF) in Response to Salinity Stress 

**Figure 6:**
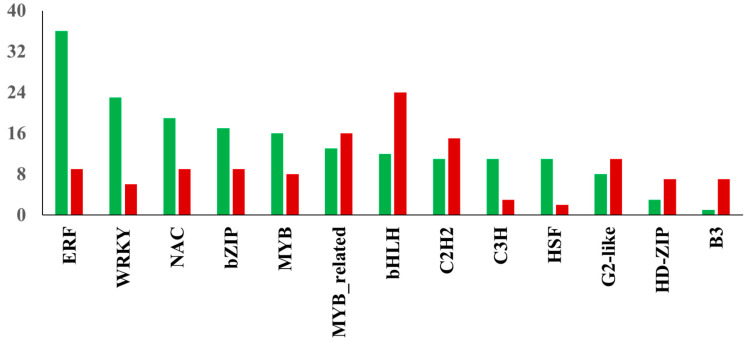
Distribution of transcription factor families. The red and green bars show the total number of down-regulated and up-regulated TFs in the respective family, respectively.

TFs play a critical role in salt stress-induced responses via transcriptional regulation of several genes in plants [22]. The assembled transcriptome demonstrated a total of 240 differentially expressed TFs were up-regulated in NaCl-treated leaf samples, while 194 TFs showed decreased expression due to salinity stress. The distribution of transcription factor families identified among DEGs in watermelon leaves in response to salt stress is shown in Figure 6. Of the up-regulated TFs, the largest number was found in ERF (36 unigenes), followed by WRKY (23 unigenes) and NAC (19 unigenes) families. In contrast, the largest number of down-regulated TFs were found in bHLH (24 unigenes), MYB-related (16 unigenes), and C2H2 (15 unigenes) families. The up-regulation of members of ERF TFs in this study indicates the significant involvement of the ethylene signaling pathway in response to salt stress in watermelon. Ethylene signaling modulates salt response via membrane receptors, components in the cytoplasm, and transcription factors [23]. The salinity stress promotes ethylene biosynthesis activating the downstream network and expression of ERFs [24]. The role of several tomato ERF.E2, ERF.F5, ERF.E3, ERF.B3, and ERF84 genes in enhancing salt tolerance has been demonstrated [25,26,27,28,29]. The ectopic expression of the barley [30], wheat [31,32], and rice [33] ERF genes also enhanced tolerance to salt stress. Consistent with our results, recent transcriptomic studies in cotton [34] and potato [35] plants revealed the induction of a high proportion of ERFs in response to salt stress, suggesting the essential role of ERFs in salt response mechanisms in plants. Despite its significance, with few exceptions [36,37], there is not much information available about the role of ERF TFs in cucurbits. The WRKY TF family is one of the largest families in higher plants and plays a crucial role in plant development and stress responses, including salt stress. Our results are consistent with a study showing the up-regulation of most WRKY genes using NaCl treatment in watermelon [38] and *Cucurbita pepo* [39]. Transcriptomic studies in other plants have demonstrated the differential expression of several members of the WRKY family in response to salt stress [40,41]. The functional role of WRKY TFs in enhancing salt tolerance has been validated using transgenic approaches by overexpressing WRKY genes from maize [42], cotton [43], soybean [44] and grapevine [45]. Similarly, NAC TFs have been implicated in a wide range of stresses, including salinity. Our data is in agreement with studies validating the induction of several NAC TFs during salt stress in watermelon [46] and melon [47]. 

#### Reliability of Transcriptome Sequencing Data

**Figure 7:**
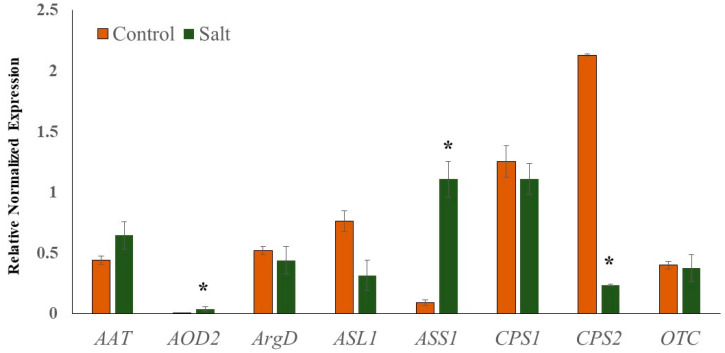
Quantitative real-time PCR (RT-qPCR) analysis.). Relative expression profiles of genes involved in the citrulline metabolic pathways in seedling leaf tissues of Crimson Sweet due to salt stress. The error bars are the means ± SE (*n* = 3), and asterisks (*) represent significant differences between treated and control tissues (*p* < 0.05).

To validate the reliability of transcriptome sequencing data, relative gene expression analysis of selected genes associated with Cit metabolism (*AAT*, N-acetylornithine; *AOD2*, N-acetylornithine deacetylase; *ArgD*, arginine decarboxylase; ASL1, arginosuccinate lyase; ASS1, arginosuccinate synthase; *CPS1* and *CPS2*, carbamoyl phosphate synthase; *OTC*, ornithine carbamoyltransferase) was performed using real-time quantitative PCR (qRT-PCR). Cit is a major non-protein amino acid in watermelon and accounts for almost 50% of the leaf amino acid pool in response to abiotic stress [19]. Cit, being an intermediate of the master metabolic pathway that synthesizes several salt stress-associated metabolites (spermine and spermidine, Pro, GABA, Arg), selected genes associated with its metabolism were expected to perturb in response to salt stress. The qRT-PCR data (Figure 7) were very consistent with the transcriptome sequencing data. Additionally, the linear regression equation y = 0.9229x − 0.395 with a high correlation (R^2^ = 0.97) showed a positive correlation and significant similarity between the two analysis techniques (Figure S2). The expression of *AOD2* and *ASS1* was significantly induced in salt-stressed samples, while the expression of *CPS2* was down-regulated. The induction in the expression of *AOD2* in salt-treated samples explains the enhanced accumulation of Cit, while the upregulation of *ASS1* supports the enhanced Arg accumulation. 

The reliability of RNA-seq data was further validated by comparing the correlations among biological replicates using the Pearson correlation coefficient (Figure S3). Unlike the high correlation among the libraries for the same treatment (i.e., biological replicates), the weak correlation across treatments (control vs. salt treatments) suggests a larger effect of salt stress on the gene expression profiles of watermelon leaf tissues. Additionally, to demonstrate the source of variance in the RNA-seq data, principal component analysis (PCA) with three principal components (PC1, 2, and 3) was performed (Figure S4). The PC score plots showed that the contribution of PC1 alone was 74.94%, followed by PC2, (9.44%), and PC3 (6.60%). The three biological replicates were collected after salt-stress and control samples were clustered together, validating the minimal variance in the analysis and suitability of data for the subsequent analysis.

## 3. Discussion

### 3.1. DEGs Associated with Endocytosis

High concentrations of salt lower the water potential and lead to ionic disequilibria across the plasma membrane, which subsequently inhibits cellular activities by entering the cytoplasm [48]. Endocytosis involves the internalization of plasma membrane proteins into the cell via a series of vesicle compartments and plays an essential role in cellular responses to environmental stimuli [49]. KEGG enrichment analysis revealed that the “Endocytosis” pathway was significantly activated in response to salt stress (Figure S5, Supplementary Materials, Table S4). Besides its role in plant growth and development, endocytosis is involved in inducing abiotic stress responses by regulating vacuolar transport [50]. Endocytosis controls cell polarity and signaling by regulating plasma membrane-associated receptors and transporters proteins [51] and the production of ROS needed for salinity tolerance [52]. The role of vesicle trafficking in adaptation against salinity stress has been validated [53,54,55,56,57,58]. Our data confirmed the induction of several *Rab* genes (ClCG02G019840, ClCG10G007150, ClCG10G012520, ClCG09G002000) in response to salt stress. The expression of native Rab7 from *Pennisetum glaucum* and its overexpression in tobacco [57] and rice [58] were greatly induced by salt stress. Exposure of plants to the salinity stress also activates phospholipase D (PLD), a phosphatidyl choline-hydrolyzing enzyme that triggers the activation of the downstream adaptive responses to relieve the damage caused by stress, including salinity [59]. Consistent with studies reporting activation of PLDs due to salt stress [60,61,62,63], expression of watermelon *PLDs* (ClCG00G000210, ClCG08G014000, ClCG06G004910) were also induced due to salt stress. Further, vacuolar protein sorting (VPS) components play an important role in maintaining osmo-homeostasis of vacuoles by sequestering toxic ions, like sodium and chloride, or other compounds involved in osmoregulation. Several VPS genes were up-regulated due to salt stress in this study. Additionally, expression of genes involved in the formation of clathrin-coated vesicles such as *clathrin* proteins (ClCG11G006070), *ADP ribosylation factors* (ClCG05G025400, ClCG07G011900, ClCG10G001580, ClCG10G021000), *ARF-guanine nucleotide exchange factors* (ClCG01G025140, ClCG11G018450, ClCG01G014890), adaptor protein (ClCG02G021820), and products in phosphatidylinositol signaling (ClCG03G009330) were up-regulated due to salt stress. The molecular chaperones *HSP70* mediate uncoating of vesicles before merging with early endosomes. In our study, four DEGs encoding *HSP70* (ClCG04G008300, ClCG09G019940, ClCG09G020000, ClCG11G011300) assigned to the endocytosis pathway were strongly up-regulated. Studies have confirmed the induction and up-regulation of HSP70 in saline stress situations rice [64], wheat [65], and potato [66].

Additionally, we found several genes in the SNARE interaction in the vascular proteins pathway were up-regulated due to salt stress (Figure S6, Supplementary Materials, Table S5). The members of the superfamily of N-ethylmaleimide-sensitive factor adaptor protein receptor (SNARE)-domain-containing proteins are involved in transport processes between individual compartments, including endocytosis [67]. Although a high number of SNARE proteins are present in the plant kingdom, their role in plant biotic and abiotic stress has only been recently understood [68]. We identified several *Syntaxin* family proteins (ClCG02G015160, ClCG07G004070, ClCG08G012870, ClCG09G005420, ClCG10G004910, ClCG10G018250) and *Golgi SNAP receptor complex member 1*, *target SNARE coiled-coil domain*, *vesicle-trafficking SEC22b*, and *vesicle transport v-SNARE 11-like* genes that were up-regulated. Several studies [69,70,71,72] have reported the functional role of SNARE interactions in salt stress, justifying the significance of SNARE proteins in salt stress-induced responses in watermelon. 

### 3.2. DEGs Related to Amino Acid Metabolism

To counter the detrimental effects of salt-induced stress, plants produce compatible solutes like free amino acids to minimize high salinity-caused osmotic stress. Positive correlations between increased salt tolerance and accumulations of total free amino acids have been reported in several crops [73,74,75,76]. 

#### 3.2.1. Branched-Chain Amino Acids (BCAAs)

Proline accumulation is known as an important mechanism in osmotic regulation in plants under a wide range of abiotic stresses [77]. However, it has been recognized that the levels of other amino acids, like BCAAs, are often greater or comparable to proline [19,78,79]. A partial deficiency of BCAAs resulted in increasing the sensitivity to salt stress [80]. Induction in the expression of *threonine dehydratase* (ClCG04G009590) along with the repression of both Thr catabolic *threonine aldolases* (ClCG02G017030 and ClCG06G009580) are in agreement with increased accumulation of BCAAs. The up-regulation of *branched-chain amino acid aminotransferase* (BCAT, ClCG08G016800), involved in both the synthesis and degradation of BCAAs, suggests its role in maintaining the non-toxic levels of free BCAAs and alleviating the injury caused by salt stress. The down-regulation of several genes involved in the degradation of BCAAs such as 2-*oxoisovalerate dehydrogenase* (ClCG03G014630), 3-*hydroxyisobutyrate dehydrogenase* (ClCG03G006140, ClCG05G009680), 3-*hydroxyisobutyryl-CoA hydrolase* (ClCG05G002680, ClCG05G016290, ClCG11G016500, ClCG03G012360), 3-*ketoacyl-CoA thiolase* (ClCG01G011180, ClCG02G002930), *methylcrotonoyl-CoA carboxylase* (ClCG02G006000), and several *aldehyde dehydrogenases* substantiates the role of BCAA accumulation during salt stress. A partial deficiency of BCAAs resulted in increasing the sensitivity to salt stress in Arabidopsis [80]. Although several studies have reported stress-induced accumulation [75], the metanalysis of transcriptome and metabolome datasets revealed that the low abundant BCAAs could also accumulate due to increased protein degradation [16]. Nevertheless, it has been suggested that BCAAs can serve either as substrates for stress-induced protein biosynthesis or as signaling molecules for regulating stress-responsive gene expression [80]. Intriguingly, *acetolactate synthase* (ALS) small subunit (ClCG03G010140), involved in BCAAs synthesis, was also down-regulated. However, though the feedback inhibition of ALS by BCAAs lacks recent experimental evidence, the non-overlapping sub-cellular localization of ALS subunits and their functional roles in Na^+^ homeostasis suggest the need for additional studies to understand the significance of ALS in salt stress-induced responses [80]. 

#### 3.2.2. Arginine-Polyamine-β-Alanine Pathway

Increased Arg accumulation, which is a precursor for polyamine synthesis, is supported by up-regulation in the expression of *argininosuccinate synthase* (ClCG06G017780), along with down-regulation of *arginine decarboxylase* (ClCG06G014050) and *arginine biosynthesis bifunctional* protein (ArgJ; ClCG10G020940), suppressing a futile cyclic version of arginine biosynthesis. Besides their role in plant growth and development, polyamines like putrescine (Put), spermine (Spm), and spermidine (Spd) play an important role in response to abiotic stress [81]. Put is synthesized directly from arginine by catabolic enzymes *arginine decarboxylase* (ADC) or *agmatine deiminase* (ADI) or from ornithine, catalyzed by *ornithine decarboxylase* (ODC). Put then converts to Spm and Spd via *spermidine synthase* and *spermine synthase* in the presence of decarboxylated S-adenosylmethionine (dcSAM) [82], which is synthesized by *SAM decarboxylase* (SAMDC) via decarboxylation of S-adenosylmethionine (SAM) [83]. In our study, RNA-seq analysis showed up-regulation of *SAMDC* (ClCG05G011880) and relatively abundant *spermidine synthases* (ClCG05G025220 and ClCG05G008800) but down-regulation of less abundant *spermidine synthases* (ClCG06G016890 and ClCG05G005220) genes. Unlike *ADC* (ClCG06G014050), expression of *ADI* (ClCG06G015290) and *ODC* (ClCG08G013990) involved in Put synthesis were also up-regulated. Although the expression of *ADC* expression was reduced, an induction of *ODC*, which catalyzes an alternative pathway of Put synthesis, seems to partly compensate the need for production of Put during salt stress. The polyamines Spm and Spd also serve as precursors of β-alanine synthesis in plants. β-alanine is converted to a quaternary ammonium osmoprotective compound called β-alanine betaine participating in tolerance to high salt concentration [84,85]. Up-regulation of *polyamine oxidase 1* (ClCG09G003930) and *polyamine oxidase 2* (ClCG07G010820, ClCG11G016630) further supports the possible involvement of β-alanine in salt-induced responses. 

#### 3.2.3. Amino Acid Transporters

Altered amino acid compositions in response to salt stress subsequently result in alterations in the expression of amino acid transporters. Several amino acid transporters have been identified in plants [86,87,88] and are grouped into two subfamilies based on sequence similarities and biochemical properties. Under salt stress, we identified a total of 45 up-regulated DEGs and 17 down-regulated DEGs associated with amino acid transport function (Supplementary Materials, Table S6). Salt stress induces changes in amino acid compositions, and the enhanced expression of amino acid transporters has been validated in Arabidopsis [89], rice [87], and wheat [90]. The DEGs involved in amino acid transport may play important roles in regulating the partitioning of different amino acids and maintain osmotic potential in response to salt stress.

### 3.3. DEGs Associated with Nitrogen Metabolism 

Nitrogen (N) metabolism, a central process for plant growth and development, is strongly influenced by salinity. Excess salt disturbs different steps of N metabolism, namely nitrate (NO_3_^-^) or ammonium (NH_4_^+^) uptake, N transport and assimilation into amino acids, and protein synthesis [91]. The absorbed N is reduced to nitrite by *nitrate reductase* (*NR*) and then to NH_4_^+^ by *nitrite reductase* (NiR). N is further assimilated into Gln and Glu via *glutamine synthetase* (*GS*) and *glutamate synthase* (*GOGAT*) and used for further biosynthesis of other nitrogenous compounds. NH_4_^+^ can be incorporated into Glu by *glutamate dehydrogenase* (*GDH*). Although the activities of GOGAT, GS, and GDH exhibit salt-dependent regulation, their regulation (induction or repression) varies among species, cultivars, tissues, and developmental changes [92,93]. A salt-induced reduction [94] and stimulation [95] of NR activity have been reported in plants. Our results showed that salt stress selectively inhibited *high-affinity nitrate transporters* (ClCG05G025540, ClCG03G003060) and *NRT1* like genes (ClCG02G009090, ClCG10G002910) but induced expression of *low-affinity nitrate transporters* (ClCG06G016390, ClCG11G002980), suggesting differential impacts of salinity on the transporters. 

Further, the down-regulation of two *glutamate dehydrogenases* (ClCG01G004910, ClCG04G005320, ClCG07G013590), *glutamine synthetase* (ClCG09G004580), and *carbonic anhydrases* (ClCG05G025330, ClCG10G018930) validated the negative impacts of salt stress on nitrogen assimilation. The expression of the gene encoding *alanine transaminase* (ClCG09G001390) was up-regulated, causing 2-oxoglutarate to generate Glu. *Glutamate decarboxylase* (*GDC*) promotes the synthesis of Pro and GABA from Glu. Our data showed that the expression of *GDCs* (ClCG00G006020, ClCG01G006890, ClCG01G006910) was highly induced in response to salt stress, implying increased accumulation of Pro and downstream metabolites such as citrulline or polyamines contribute towards salt tolerance. Differential responses of various members of the same gene family due to salt stress are consistent with a study in rice [96]. Accumulation of excess Ser can be attributed to the activation of phosphorylated pathways of Ser synthesis as the expression of *D-glycerate 3-kinase* (ClCG09G002370), *D-3-phosphoglycerate dehydrogenase* (ClCG05G010250) and *phosphoserine aminotransferase* (ClCG10G000330) were strongly up-regulated. Ser is considered as a critical player in biochemical responses for the regulation of intracellular redox, energy levels, and cellular pH, particularly in stress conditions [97]. 

### 3.4. Disruption of the Energy Metabolisms by the Salt Stresses 

Effective photosynthesis results through coordinated activities of four protein complexes—PSI, PSII, the cytochrome b6/f complex, and ATP synthase. As confirmed in the GO and KEGG pathway enrichment analysis, several DEGs identified in this study associated with these complexes were down-regulated due to salt stress (Figure S7, Supplementary Materials, Table S7). The down-regulation of PsbO (ClCG01G016370), PsbP (ClCG07G010800), PsbQ (ClCG05G000900, ClCG03G005130), PsbS (ClCG08G005640), and PsbW (ClCG02G016710, ClCG09G007590) in PSII complex were consistent with decreasing magnitude of Fv/Fm, suggesting impaired chlorophyll fluorescence of PSII during salt stress progression. Although both the PSI and PSII reaction centers are affected by salt stress, studies in cucumber found that PSI is more vulnerable to injury than PSII [98]. Several genes encoding PSI protein complex viz PsaE (ClCG11G010230), PsaF (ClCG01G009000), PsaG (ClCG10G004510), PsaH (ClCG07G013150), PsaK(ClCG01G025030), PsaL(ClCG11G010740), PsaN (ClCG01G015380), and PsaO (ClCG01G011760), along with proteins involved in photosynthetic electron transfer (PetE, PetF, PetH), were also down-regulated confirming inhibition of photosynthetic activities under salt stress. A decrease in electron transfer rate accumulates excess electrons leading to electron leakage, which results in the outbreak of reactive oxygen species (ROS) and damage to the PSII reaction center [99,100]. Further, nearly all the proteins (Lhca1 to Lhca5, Lhcb1 to 4, Lhcb7) involved in light-harvesting chlorophyll (LHC) were down-regulated (Figure S8, Supplementary Materials, Table S7). The inactivation of photosynthesis and LHC complex due to salt stress observed in this study is consistent with several studies [56,101,102] and validates the role of PSI and PSII complexes in balancing energy supply and ROS generation under salt stress in watermelon. 

### 3.5. DEGs Associated with Hormonal Regulation

**Figure 8:**
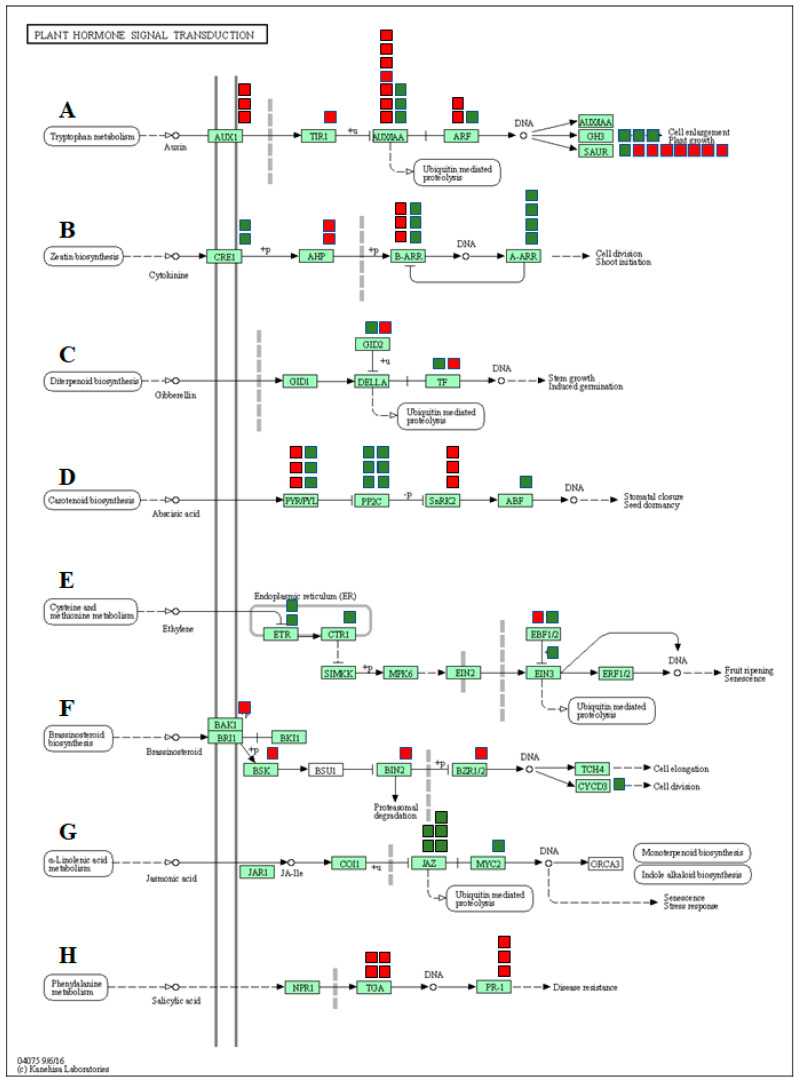
DEGs mapped to the plant hormone signaling transduction pathways in watermelon leaves. (**A**) Auxin signaling pathway, (**B**) cytokinin pathway, (**C**) gibberellin (GA) pathway, (**D**) abscisic acid pathway, (**E**) ethylene signaling pathway, (**F**) brassinosteroid pathway, (**G**) jasmonic acid pathway, (**H**) salicylic acid pathway. The red and green boxes show the number of down- or up-regulated DEGs, respectively.

In the present study, the functional analysis identified many DEGs associated with hormone signaling transduction pathways emphasizing the involvement of plant hormones in regulating the response to salt stress in watermelon leaves. We grouped the DEGs into various phytohormone signaling pathways, such as auxin (AUX), cytokinin (CTK), gibberellin (GA), abscisic acid (ABA), ethylene (ETH), brassinosteroid (BR), jasmonic acid (JA), and salicylic acid (SA) (Figure 8, Supplementary Materials, Table S8). Studies have confirmed the reduced auxin levels and decreased auxin transporter expression in plants under saline conditions [103,104]. The expression of most of the genes involved in auxin signal transduction pathways such as auxin transporter protein 1 (AUX1), transport inhibitor response (TIR1), auxin response factor gene (ARF), auxin early response gene (Aux/IAA), and small auxin-up RNAs (SAUR) were significantly down-regulated in response to salt stress. Up-regulation of GH3 genes due to salt stress is consistent with previous studies [105] and is possibly responsible for triggering cellular mechanisms to protect cell auxin homeostasis during changes in extracellular auxin levels. Although cytokinins play an important role in plant growth and development, numerous pieces of evidence indicate both positive and negative effects on stress tolerance. Salt-treated plants showed increased or decreased accumulation of active cytokinins in plants [106,107,108]. In our study, expression of genes CRE1, B-ARR, and A-ARR were up-regulated, while B-ARR was down-regulated due to salt stress. The changes in the expression of genes associated with cytokinins are consistent with salt-induced changes in tomato [109,110] and Arabidopsis [108] plants. 

The ABA signaling pathway is associated with salt stress-induced responses and helps plants by reducing the buildup of Na+ and improving osmotic adjustment. In the present study, three of the ABA receptors (PYR/PYL) and serine/threonine-protein kinase 2 (SNRK2) were significantly down-regulated in salt treatment, suggesting inhibition of the ABA signaling pathway by saline treatments. The only salt-induced changes in the expression of two F-box gibberellin-insensitive dwarf2 (GID2) and transcription factor genes associated with the GA signaling pathway suggest a sub-optimal role of the GA pathway in salt stress-induced responses in watermelon. 

Although ethylene is a stress hormone regulating numerous stress responses, its specific roles in salt stress tolerance in plants remain unclear [111]. In this study, we observed up-regulation of ethylene receptors (ETR) and serine/threonine-protein kinase CTR1 (CTR1), which serve as negative regulators of the ethylene signaling transduction pathway. On the contrary, a positive regulator of ethylene signal transduction, ethylene-insensitive protein 3 (EIN3), was also up-regulated. No significant differences in expression levels of EIN2 and ERF1/2 genes were observed. Taken together, salt stress seems to have either a negative or trivial impact on the ethylene signaling pathway. 

In the present study, genes involved in the BR pathway viz. BRI1-associated receptor kinase (BAK1), BR-signaling kinase (BSK), brassinosteroid insensitive 2 (BIN2), and brassinosteroid resistant 1/2 (BZR1/2) were significantly up-regulated, suggesting activation of the BR signaling pathway in response to salt stress. Similar activation of genes involved in the BR signaling pathway during salt stress has been reported in Arabidopsis [112]. Up-regulation of the cyclin gene CycD3 in our study is consistent with a study showing similar responses due to the exogenous application of 24-EBR [113]. Although the results confirm the activation of the BR signaling pathway, the exact functional relevance of the BR pathway in salt-induced responses needs further investigation. 

In the JA signal pathway, the expression of jasmonate ZIM domain-containing protein (JAZ) genes and JASMONATE INSENSITIVE 1/MYC2 (JIN1/MYC2) were significantly up-regulated due to salinity, indicating activation of JA signaling transduction by saline stress. The up-regulation of COI1-dependent JA-responsive JAZ genes due to salt stress has been reported in Arabidopsis [114]. It is plausible to assume that salt stress-induced JA-Ile promotes the interaction of JAZ proteins with COI1, followed by their degradation via the 26S proteasome and de-repression of MYC2 to induce transcription of JA-responsive genes in watermelon.

## 4. Materials and Methods 

### 4.1. Salt Stress Experiment and Photochemical Efficiency Measurement

Watermelon (*C. lanatus* L. cv. Crimson Sweet) seeds were sown in trays filled with a soilless media (Quick Dry Infield Conditioner, Turface Atheletics™, Buffalo Grove, IL, USA) and placed in the greenhouse at the Texas A&M AgriLife Research and Extension Center, Uvalde, TX, USA. Six-week-old seedlings with three fully expanded leaves were carefully lifted out from Turface media, washed under running water, and incubated in 50 mL tubes (VWR^®^, Radnor Corporate Center, Radnor Township, PA, USA) containing 300 mM NaCl and deionized water as a control (Barnstead™ Smart2Pure™ Water Purification System, Thermo Scientific, Waltham, MA, USA). The concentration of NaCl used in this study was previously validated for its sensitivity in watermelon [115,116]. Chlorophyll fluorescence was measured at the end of the experiment using a portable fluorometer (PAR-FluorPen FP 110/D; PSI (Photon Systems Instruments), Drasov, Czech Republic) after dark adaption for 30 min. This measurement was initiated from 1 h after initiation of the treatment and was continued every 2 h. The maximum photochemical efficiency of PSII (Fv/Fm) was calculated according to the manufacturer’s protocol. After 7 h of exposure to salt stress, leaf and root tissue samples were collected from four independent seedlings for each treatment and flash-frozen using liquid nitrogen before storing at −80 ℃ for further processing.

### 4.2. Extraction Method and Quantification of Free Amino Acids with Ultra-Performance Liquid Chromatography-Electron Spray Ionization Tandem Mass Spectrometry (UPLC-ESI-MS/MS)

Approximately 20 mg frozen tissue samples collected into 2 mL microcentrifuge tubes were homogenized into fine powder in a paint shaker (Harbil model 5G-HD paint shaker) using 3 mm stainless steel beads (Demag stainless steel balls, Abbott Ball Company, Inc., West Hartford, CT, USA) to quantify free amino acids. Amino acids were extracted using an established protocol [34] by suspending the homogenized samples in 100 mM cold HCl extraction buffer, followed by incubation on ice (~20 min) and then centrifuging at a speed of 14,609× *g* for 20 min at 4 °C. The supernatants were collected and filtered through a 96-well 0.45-μm-pore filter plate (Pall^®^ Life Sciences Filter, Pall Corporation, Port Washington, NY, USA). The eluents collected in 96-well trap plates were stored at −20 °C for further amino acid quantification. 

The derivatization of filtrates was carried out with an AccQ•Tag 3X Ultra-Fluor™ derivatization kit (Waters Corporation, Milford, MA, USA) following the standard protocol. L-Norvaline (Sigma, St. Louis, MO, USA) was used as an internal standard at a fixed concentration of at 25 pmol/µl. Amino acid calibrators were purchased from Kairos^TM^ Amino Acid Kit (Waters Corporation, Milford, MA, USA). Lyophilized amino acid powder of six amino acid calibrators representing different concentrations (5.0 pmol/µL to 1000 pmol/µL) was reconstituted with 2 mL of 0.1 M HCl to establish detection limits ranging from 0.45 pmol/µL to 90 pmol/µL. Calibration curves were built in TargetLynxTM Application Manager (Waters Corporation, Milford, MA, USA).

UPLC-ESI-MS/MS analysis was performed using a Waters Acquity H-class UPLC system equipped with a Waters Xevo TQ mass spectrometer by using electrospray ionization (ESI) probe. The Waters Acquity H-class UPLC system was composed of an autosampler, a binary solvent manager, a Waters^®^ ACQUITY UPLC^®^ Fluorescence (FLR) detector, a column heater and a Water’s AccQ•Tag Ultra column (2.1 mm i.d. × 140 mm, 1.7 μm particles). The mobile phase consists of water phase (A) (0.1% formic acid *v*/*v*) and acetonitrile (B) (0.1% formic acid *v*/*v*) with a stable flow rate at 0.5 mL/min and column temperature setting at 55 ℃. The gradient of non-linear separation was set as follows: 0–1 min (99% A), 3.2 min (87.0% A), 8 min (86.5% A), and 9 min (5% A). Finally, 2 μL of the derivatized sample was injected onto the column for analysis. IntelliStart software (Waters Corp, Milford, MA, USA) was used to optimize amino acid multiple reaction monitoring (MRM) transitions, collision energy values, and cone voltage. The ESI source was set to 150 °C with the gas desolvation flow rate at 1000 L/h, gas flow cone at 20 L/h, desolvation temperature at 500 °C, the capillary voltage at 2.0 kV, gas collision energy varied from 15 to 30 V, and cone voltage at 30 V for detecting all amino acids. MRM was operated in positive mode. Water’s MassLynx™ 4.1 software was used for instrument monitoring and data acquisition. The data integration, calibration curves, and quantitation (0.45–90 pmol/µL) were carried out with TargetLynx™ Application Manager (Waters Corporation, Milford, MA, USA).

### 4.3. cDNA Library Preparation and RNA-Seq Analysis of Salt-Stressed Seedling Leaves

Six independent libraries were created by using a total of 6 RNA samples from 3 replicate leaf tissues of cv. Crimson Sweet under the control and salt-treated condition. The samples were flash-frozen in liquid nitrogen and ground to a fine powder using 3-mm-diameter steel balls (Abbott Ball, West Hartford, CT, USA) in a paint shaker (Harbil, Wheeling, IL, USA). Total RNA was extracted using an RNeasy^®^ Plant Mini Kit (QIAGEN Sciences, Germantown, MD, USA) as per the manufacturer’s protocol. The purity of the RNA was confirmed using a NanoPhotometer^®^ spectrophotometer (IMPLEN GmbH, Inc., München, Germany). The RNA Nano 6000 Assay Kit of the Bioanalyzer 2100 system (Agilent Technologies, Santa Clara, CA, USA) was used to assess RNA integrity and quantitation. Sequencing libraries were generated using a NEBNext^®^ Ultra™ RNA Library Prep Kit for Illumina^®^ (New England Biolabs, Ipswich, MA USA) following the manufacturer’s protocol. The clustering of the index-coded samples was performed on a cBot Cluster Generation System using PE Cluster Kit cBot-HS (Illumina) according to the manufacturer’s instructions. After cluster generation, the libraries were sequenced on an Illumina Hiseq platform, and 150 bp paired-end reads were generated. Raw reads of fastq format were processed to obtain clean reads by removing the adapter, reads containing poly N (reads when uncertain nucleotides constitute more than 10 percent of either read; N > 10%), and low-quality reads (reads when low-quality nucleotides (base quality less than 20) constitute more than 50% of the read). The Qscore (quality value) of over 50% bases of these reads is ≤5) from raw data. At the same time, Q20, Q30, and GC content of the clean data were calculated. Watermelon reference genome version 2 (cv. Charleston Gray) and gene model annotation files were downloaded from CuGenDB (http://cucurbitgenomics.org/). Index of the reference genome was built using Bowtie v2.2.3, and paired-end clean reads were aligned to the reference genome using TopHat v2.0.12. HTSeq v0.6.1 was used to count the reads mapped to each gene. FPKM [117] of each gene was calculated based on the length of the gene and reads count mapped to this gene. Differential expression analysis of genes was performed using the DESeq R package (1.18.0) [118]. Genes with *p*-value < 0.05 found by DESeq were considered as differentially expressed. Gene ontology (GO) [119] enrichment analysis of differentially expressed genes was implemented using the GOseq R package, in which gene length bias was corrected. GO terms with a *p*-value less than 0.05 were considered significantly enriched by DEGs. To test the statistical enrichment of differential expression genes, KOBAS software in the Kyoto Encyclopedia of Genes and Genomes (KEGG) pathways database [120] was used. To identify the source of variance in the expressed transcripts between control and salt treatment, and the repeatability of samples within a group, principal component analysis (PCA) was used. PCA was performed using the scikit-learn package [121] and plotted using Matplotlib [122]. The RNA-seq dataset is accessible through GEO Series accession number GSE146087 (https://www.ncbi.nlm.nih.gov/geo/). 

### 4.4. Validation by Quantitative Real-Time PCR

To validate the RNA-seq data, total RNA was extracted from three replicate leaf tissues of salt-stressed seedlings (Crimson Sweet). The expression pattern of selected DEGs was examined using quantitative real-time PCR (RT-qPCR). The gene-specific primers based on the selected unigene sequences (Table S9) were designed using Primer Premier 3.0 software. Total RNA was extracted with the Quick-RNA™ Miniprep Kit (Zymo Research Corporation, Irvine, CA, USA) followed by DNase1 (Zymo Research Corporation, Irvine, CA, USA) treatment, and subjected to reverse transcription using iScript RT Supermix (Bio-Rad Laboratories, Inc., Hercules, CA, USA ). The quality and quantity of the RNA were examined using a Denovix DS-11+ spectrophotometer (DeNovix Inc. Wilmington, DE, USA). Gene expression analysis via reverse transcription-qPCR was performed using a BioRad CFX96 qPCR instrument and by using a SsoAdv Univer SYBR GRN Master Kit (Bio-Rad Laboratories, Inc., Hercules, CA, USA). Watermelon β-actin and α-tubulin5 genes [123] were used as the internal controls, and the relative expression levels (Cq values) for each gene were normalized by taking an average of three biological replicates. The relative expression levels of each gene were calculated using the 2^−ΔΔCt^ method. The primers for qPCR used in this chapter are listed in Supplementary Materials, Table S9.

## 5. Conclusions

In conclusion, this study represents comprehensive information regarding the transcriptome of watermelon seedlings in response to salt stress. The transcriptome profiling generated over 43 million reads from the control and salinity-treated libraries. The transcriptome assembly detected 7622 genes that were expressed differentially in response to salinity. These differentially expressed genes included transcription factors, genes related to primary metabolism, endocytosis, hormonal pathways, and transporters involved in responses to salinity. The gene expression patterns of the TFs identified in this study help in improving our understanding of the significance of transcriptional regulation in watermelon during salt stress. These results provide a basis for future studies aimed at discovering novel genes, their functional validation in model species, and finding molecular mechanisms associated with salt tolerance in watermelon. 

## Supplementary Materials

The following are available online at https://www.mdpi.com/1422-0067/21/17/6036/s1.

## Abbreviations

GO	Gene Ontology
KEGG	Kyoto Encyclopedia of Genes and Genomes
