# Supplementary figures and images for: Transcriptomic Analysis of Short-Term Salt Stress Response in Watermelon Seedlings
Source: Int J Mol Sci. 2020 Aug 21;21(17):6036. doi: 10.3390/ijms21176036 (PMC7504276; doi:10.3390/ijms21176036)

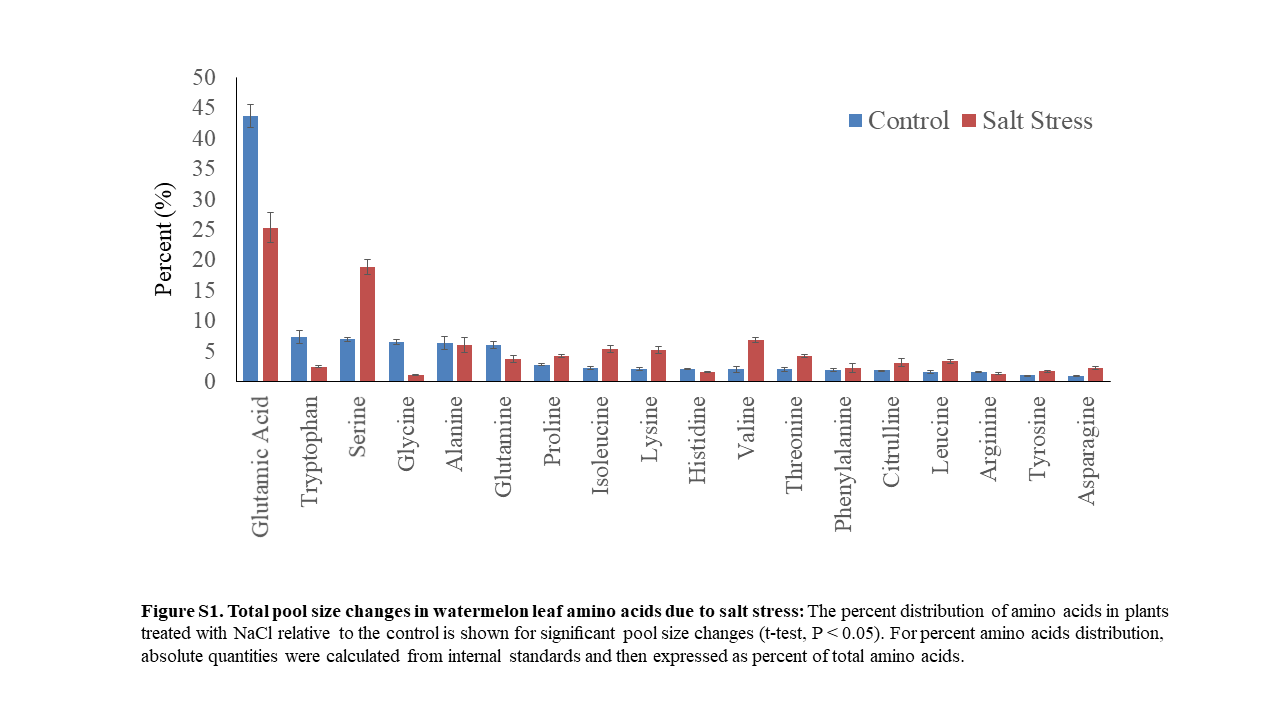

Supplement: Supplementary file 1 [file ijms-21-06036-s001.zip › Supplementary for conversion/Figure S1.TIF]

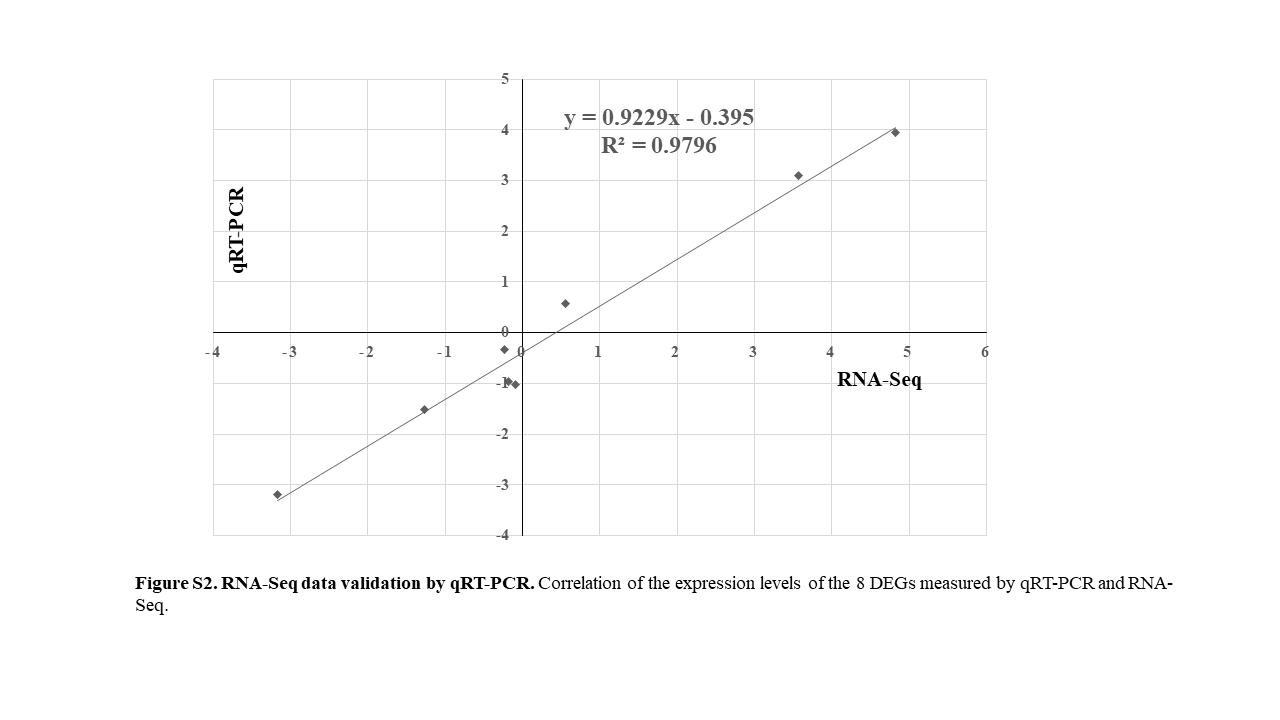

Supplement: Supplementary file 1 [file ijms-21-06036-s001.zip › Supplementary for conversion/Figure S2.TIF]

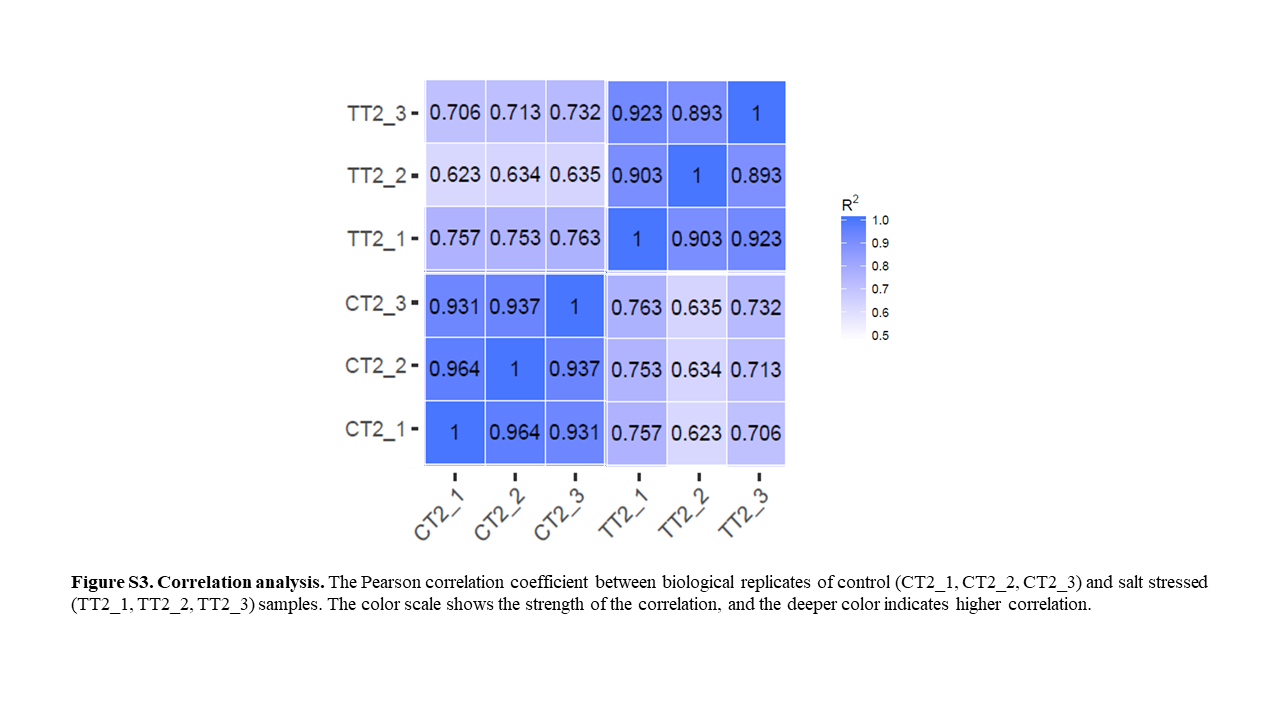

Supplement: Supplementary file 1 [file ijms-21-06036-s001.zip › Supplementary for conversion/Figure S3.TIF]

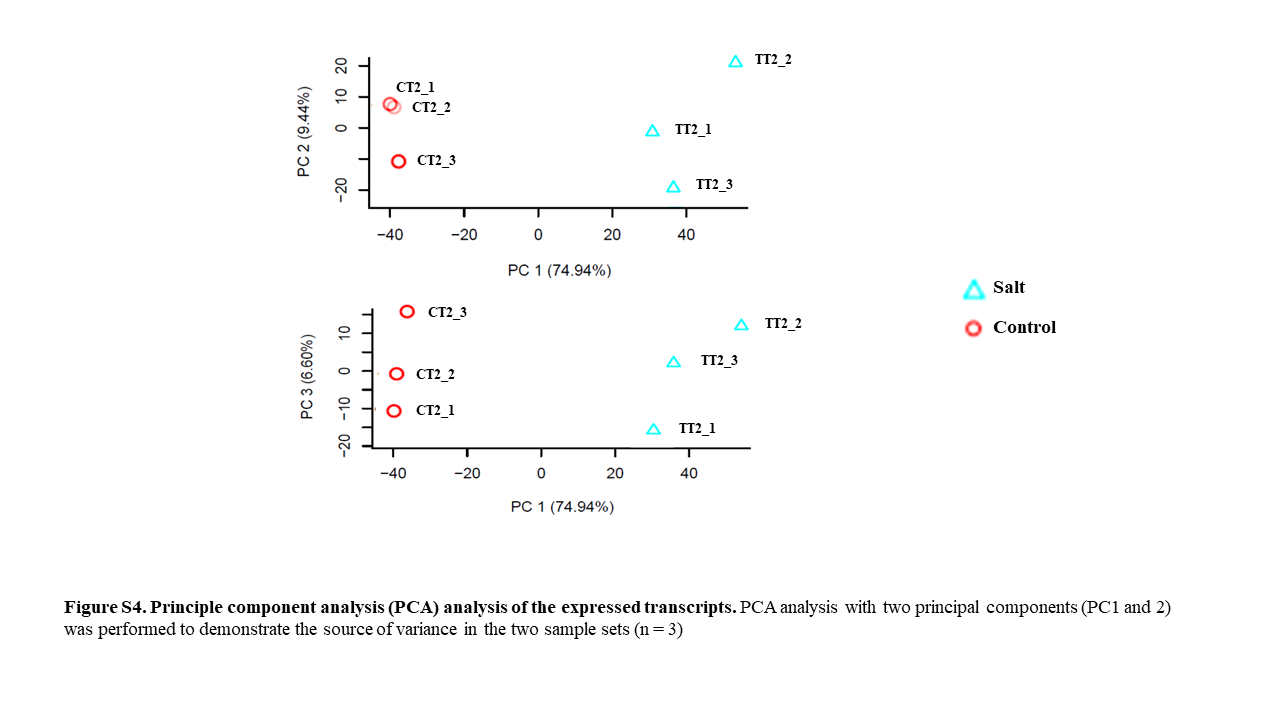

Supplement: Supplementary file 1 [file ijms-21-06036-s001.zip › Supplementary for conversion/Figure S4.TIF]

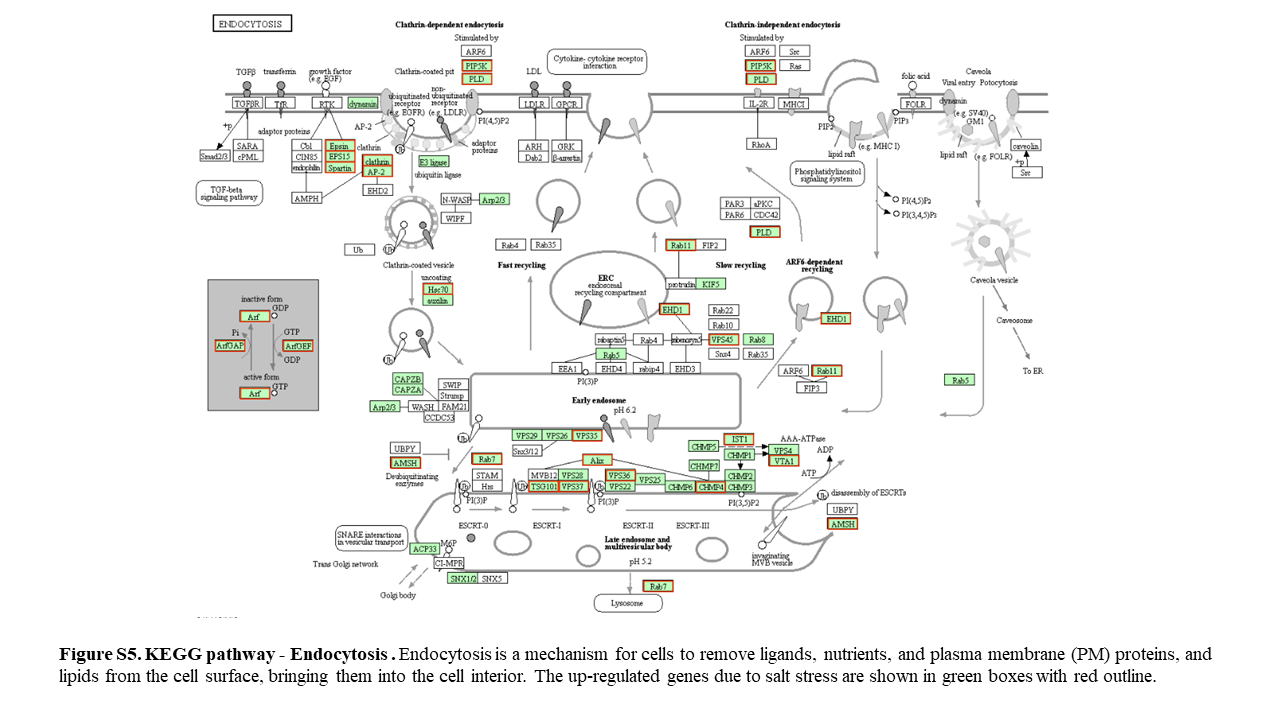

Supplement: Supplementary file 1 [file ijms-21-06036-s001.zip › Supplementary for conversion/Figure S5.TIF]

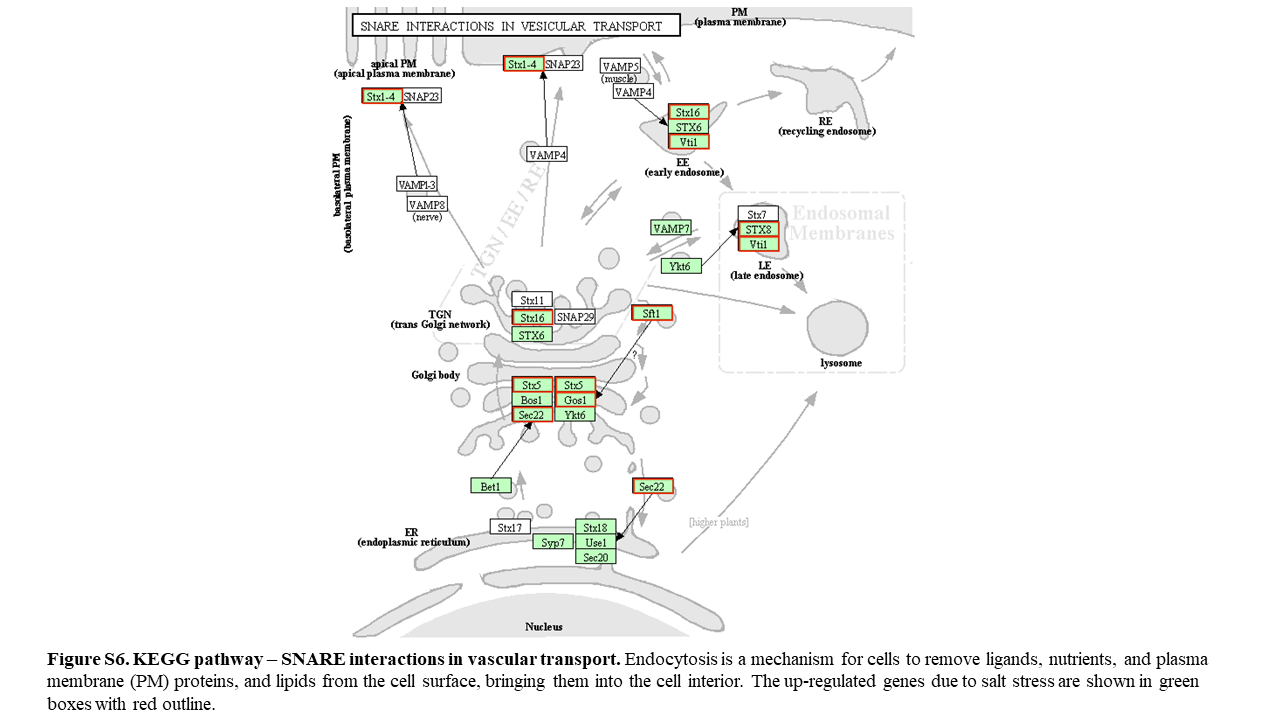

Supplement: Supplementary file 1 [file ijms-21-06036-s001.zip › Supplementary for conversion/Figure S6.TIF]

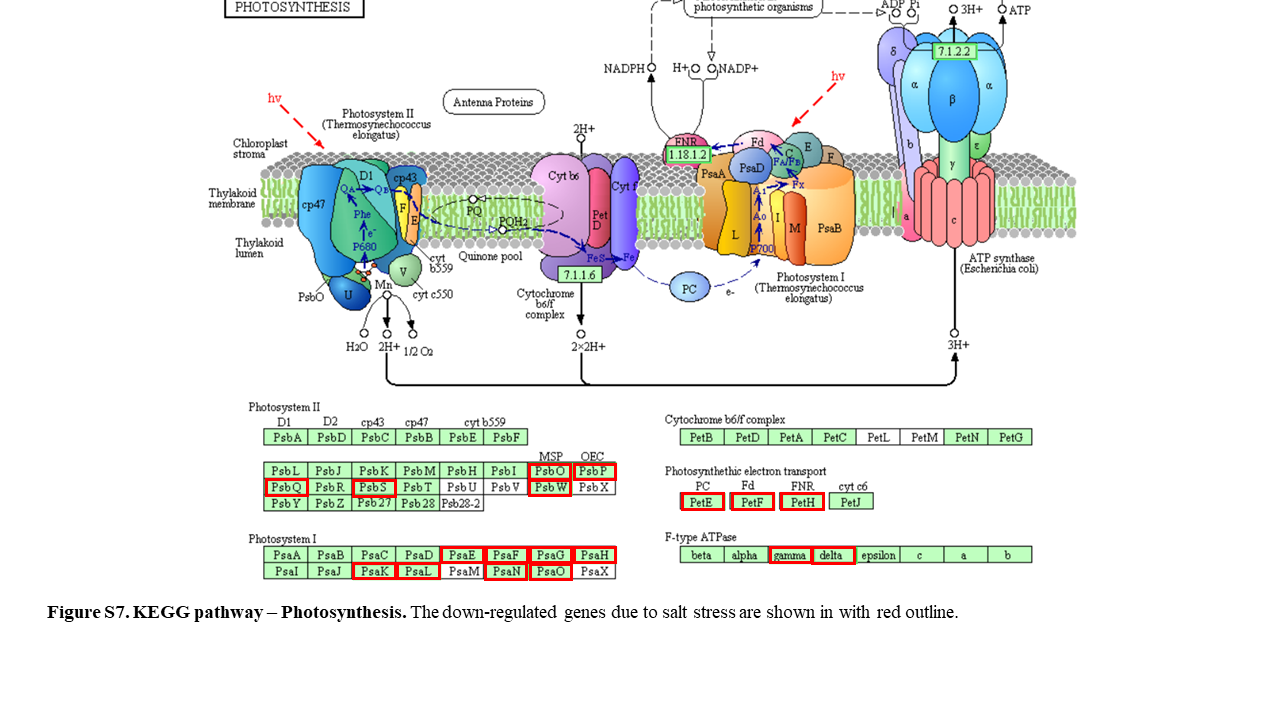

Supplement: Supplementary file 1 [file ijms-21-06036-s001.zip › Supplementary for conversion/Figure S7.TIF]

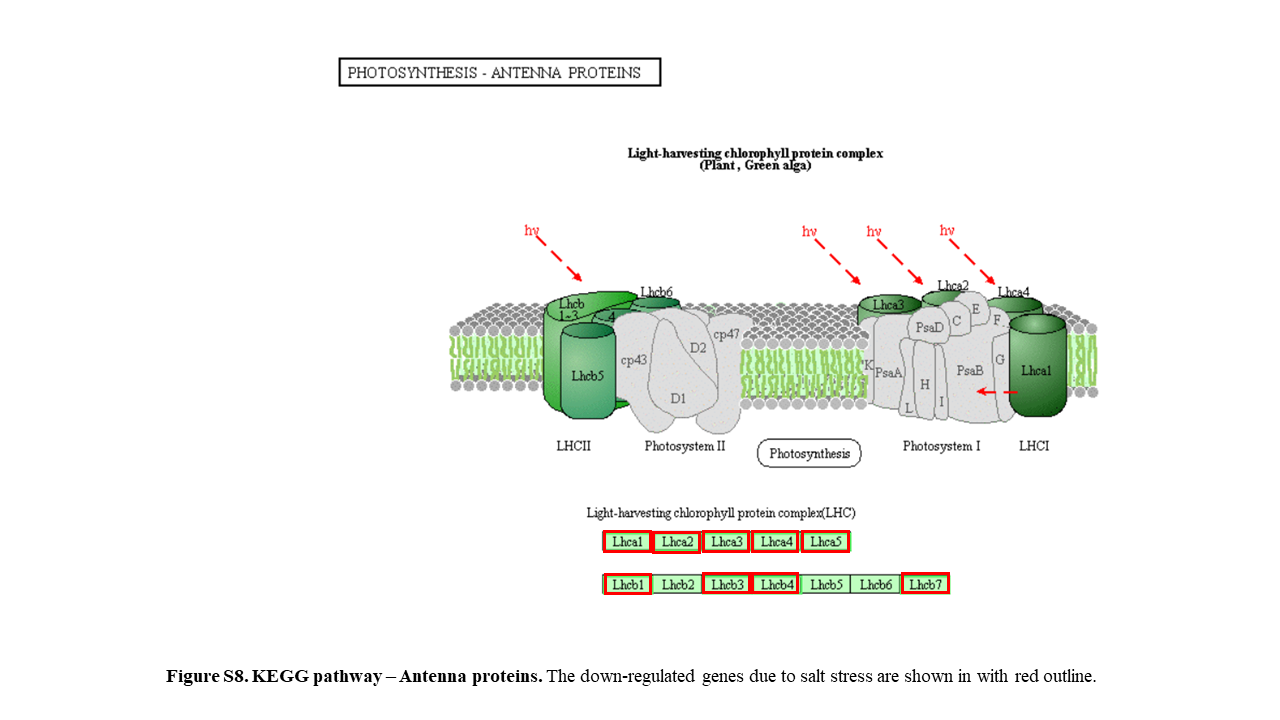

Supplement: Supplementary file 1 [file ijms-21-06036-s001.zip › Supplementary for conversion/Figure S8.TIF]

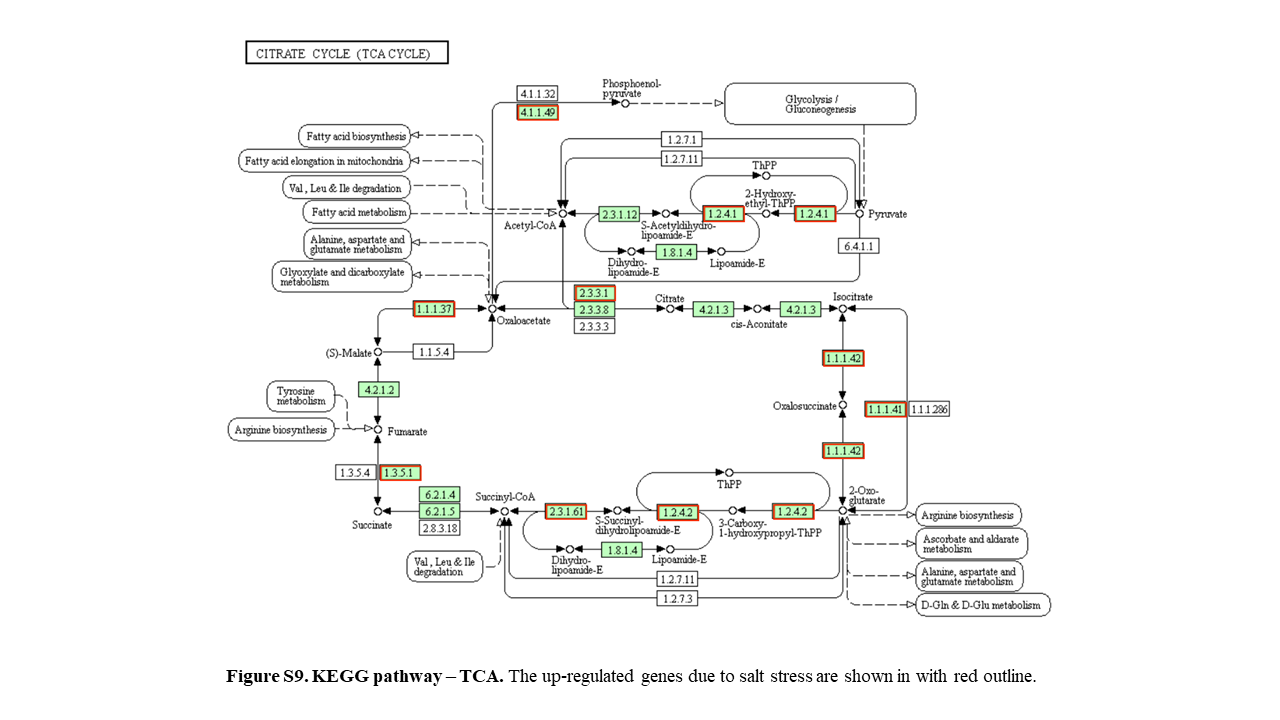

Supplement: Supplementary file 1 [file ijms-21-06036-s001.zip › Supplementary for conversion/Figure S9.TIF]
